# Randomized, double-blind, placebo-controlled pilot study of metformin as an adjunctive therapy in Parkinson’s disease

**DOI:** 10.3389/fphar.2025.1497261

**Published:** 2025-05-02

**Authors:** Hayam Ali AlRasheed, Mostafa M. Bahaa, Thanaa A. Elmasry, Eman I. Elberri, Fedaa A. Kotkata, Ramy M. El Sabaa, Yasmine M. Elmorsi, Mostafa M. Kamel, Walaa A. Negm, Amir O. Hamouda, Khlood Mohammad Aldossary, Muhammed M. Salahuddin, Mohamed Yasser, Mamdouh Eldesouqui, Manal A. Hamouda, Nashwa Eltantawy, Mirna E. Elawady, Mahmoud S. Abdallah

**Affiliations:** ^1^ Department of Pharmacy Practice, College of Pharmacy, Princess Nourah bint Abdulrahman University, Riyadh, Saudi Arabia; ^2^ Pharmacy Practice Department, Faculty of Pharmacy, Horus University, New Damietta, Egypt; ^3^ Pharmacology and Toxicology Department, Faculty of Pharmacy, Tanta University, Tanta, Al-Gharbia, Egypt; ^4^ Pharmacy Practice Department, Faculty of Pharmacy, Sinai University, Arish Branch, Arish, Egypt; ^5^ Department of Clinical Pharmacy, Faculty of Pharmacy, Tanta University, Tanta, Al-Gharbia, Egypt; ^6^ Department of Clinical Pharmacy, Faculty of Pharmacy, Menoufia University, Shebin El-Kom, Menoufia, Egypt; ^7^ Psychiatry Department, Faculty of Medicine, Tanta University, Egypt; ^8^ Pharmacognosy Department, Faculty of Pharmacy, Tanta University, Tanta, Al-Gharbia, Egypt; ^9^ Department of Biochemistry and Pharmacology, Faculty of Pharmacy, Horus University, New Damietta, Egypt; ^10^ Department of Pharmaceutics, Faculty of Pharmacy, Port Said University, Port Said, Egypt; ^11^ Department of Pharmaceutics, Faculty of Pharmacy, East Port Said National University, Port Said, Egypt; ^12^ Department of Pharmaceutics and Industrial Pharmacy, Faculty of Pharmacy, Horus University, New Damietta, Egypt; ^13^ Department of Basic Medical Sciences, College of Medicine, AlMaarefa University, Riyadh, Saudi Arabia; ^14^ Department of Pharmacy Practice, Faculty of Pharmacy and Drug Technology, Egyptian Chinese University, Cairo, Egypt; ^15^ Department of Pharmacy Practice, Faculty of Pharmacy, Sinai University, Kantara, Ismailia, Egypt; ^16^ Department of Clinical Pharmacy, Faculty of Pharmacy, University of Sadat City (USC), Sadat City, Menoufia, Egypt; ^17^ Department of PharmD, Faculty of Pharmacy, Jadara University, Irbid, Jordan

**Keywords:** Parkinson disease, metformin, neuro-inflammation, α-synuclein, TLR-4

## Abstract

**Background:**

Parkinson’s disease (PD) is caused by the progressive loss of dopaminergic neurons in the substantia nigra. Neuroinflammation is considered a key factor contributing to the pathophysiology of PD. Current gold-standard therapies for PD provide only symptomatic relief without slowing disease progression, highlighting the need to develop new disease-modifying treatments. Metformin has been demonstrated to exert a neuroprotective role in several neurodegenerative disorders including PD.

**Aim:**

This study aimed to clarify the role of metformin as adjuvant therapy in patients with PD.

**Methods:**

Sixty patients with PD were divided into 2 groups (n = 30). Patients in group 1 received levodopa/carbidopa (250/25 mg) three times daily for 3 months plus placebo (Control group), while those in group 2 received levodopa/carbidopa (250/25 mg) three times daily and 500 mg metformin two times daily (Metformin group). Patients were assessed via Unified Parkinson’s Disease Rating Scale (UPDRS). The serum concentrations of toll like receptor 4 (TLR-4), α-synuclein, brain derived neurotropic factor (BDNF), and high mobility group box 1 (HMGB-1) were measured before and after treatment.

**Primary outcome:**

The improvement in UPDRS from baseline to 3 months.

**Secondary outcome:**

Change in the level of biological markers.

**Results:**

The control group did not show significant difference in UPDRS when compared to their baseline value by Wilcoxon test (*P* > 0.05), meanwhile the metformin group showed significant difference when compared to before treatment by Wilcoxon test (*P* < 0.05). There were no significant differences between the two groups in UPDRS after treatment (*P* > 0.05) by Man Whitney test. However, the metformin group showed a significant decrease in TLR-4, HMGB-1, and α-synuclein along with a statistically significant increase in BDNF (*P* < 0.05) when compared to its baseline and control group. The control group did not show any significant changes in all markers when compared to their baseline.

**Conclusion:**

While no significant differences in UPDRS scores were observed between the metformin and control groups, trends in biomarker changes suggest a potential impact of adjunctive metformin use on the underlying pathophysiology of PD. Further studies are needed to assess its effects on motor symptoms over a longer duration.

**Clinical Trial Registration:**

identifier NCT05781711.

## 1 Introduction

Parkinson's disease (PD) is the second most common and and fastest-growing neurodegenerative disorder worldwide (Dorsey et al., 2018; Badawoud et al., 2024). The primary clinical manifestations of PD are motor symptoms, which have been attributed to the selective loss of dopaminergic neurons in the substantia nigra pars compacta (SNpc) (Cherian and Divya, 2020; Al-Kuraishy et al., 2024). The formation of intracellular proteinaceous aggregates, known as Lewy bodies—primarily composed of α-synuclein (α-Syn) —in surviving neurons is another hallmark of PD. Studies have shown that α-synuclein aggregates can induce neuronal toxicity, leading to neuronal death through various mechanisms (Cherian and Divya, 2020; Alrouji et al., 2024a; Alrouji et al., 2024b). The aggregation of α-Syn plays a pivotal role in the pathogenesis of PD and other synucleinopathies. α-Syn is a lysine-rich, soluble, and amphipathic protein that is predominantly expressed in neurons (Serratos et al., 2022; Turkistani et al., 2024). Pathogenic mechanisms affecting the structural and functional stability of α-Syn—including endoplasmic reticulum stress, Golgi complex fragmentation, dysfunctional protein degradation systems, aberrant interactions with mitochondrial membranes and nuclear DNA, altered cytoskeleton dynamics, disruption of the neuronal plasma membrane, impaired vesicular transport, and the formation of extracellular toxic aggregates—contribute to the progression of PD and other synucleinopathies (Serratos et al., 2022; Turkistani et al., 2024).

Both genetic and environmental factors play significant roles in PD risk, with genetic factors accounting for approximately 10%–15% of cases and 5%–10% have a monogenic form of the disease with Mendelian inheritance (Bogers et al., 2023; Deng et al., 2018). Neuroinflammation is considered a key factor significantly influencing the pathophysiology of PD. High mobility group box-1 (HMGB1) protein has been identified as a potential inflammatory biomarker in PD (Gan et al., 2020). Targeting key cell receptors, including advanced glycation end products (AGE) and Toll-like receptor 4 (TLR-4), mediates immune responses primarily through the stimulation of endothelial cells and macrophages (Cross et al., 2024). HMGB1 in the nucleus is translocated to extracellular target cells via passive and active release, where it interacts with the receptor for AGE. (Yuan et al., 2024). AGE is expressed on endothelial cells, monocytes, macrophages, and other cells surfaces. After combining with HMGB1, it mediated the activation of nuclear factor kappa-B (NF-kB), janus kinases (JAK), signal transducer and activator of transcription factor (STAT), and mitogen activated protein kinase (MAPK) family (Herold et al., 2007). The main receptors for HMGB1 on the surface of macrophages are TLR-2 and TLR-4, with TLR-4 playing a crucial role in neurodegenerative diseases (Paudel et al., 2020a). HMGB1 expression promotes the activation of astrocyte AGE–mitogen activated protein kinase (MAPK) signaling, which in turn promotes the expression of chemokines, cyclooxygenase 2 (COX-2), matrix metalloproteinase 9, and many other bioactive molecules especially those involved in neuroinflammation (Karuppagounder et al., 2014). A previous study showed that HMGB1 induced the expression of interleukin (IL) and other inflammatory cytokines in brain tissues (Tang et al., 2022). Expression of this neuroinflammatory cytokine promotes neuron apoptosis and increases the development and progression of neurodegenerative disease in the central nervous system (Zhang et al., 2023). HMGB1 also regulated the release of excitatory neurotransmitters (Lin et al., 2020). It was suggested that HMGB1 also promotes the release of endogenous glutamic acid and D-aspartic acid *in vitro* from glial cells (Dai et al., 2021). In-depth research on HMGB1 has shown that HMGB1 is associated with TLR-4-mediated inflammatory response and a variety of diseases, such as sepsis, gliomas, and PD (Yang et al., 2018a). The HMGB1–TLR-4 axis is key to the inflammatory response; damaged cells and activated macrophages actively or passively release HMGB1, which induces the secretion of tumor necrosis factor-α (TNF-α), IL-6, and other inflammatory cytokines through signaling pathways. Early proinflammatory factors and HMGB1 itself promote the release of HMGB1 to form a loop, which amplifies the inflammatory response (Wang et al., 2022).

Metformin, a member of the biguanide family commonly used to treat type 2 diabetes, appears to both reduce hepatic glucose production and enhance insulin sensitivity in the liver and peripheral tissues (Alrouji et al., 2023). Metformin is widely recognized as an adenosine monophosphate-activated protein kinase (AMPK) stimulator, potentially accelerating AMPK phosphorylation at the Thr172 residue (Alrouji et al., 2023). Notably, metformin is an effective treatment for PD, significantly reducing dopaminergic neuron death and enhancing antioxidant activity (Ordovich-Clarkson et al., 2024). The neuroprotective potential of metformin has been investigated based on emerging evidence from preclinical and clinical studies (Paudel et al., 2020b; Roberts et al., 2024; Vassal et al., 2024). Regarding the underlying molecular mechanisms, metformin has been shown to inhibit α-syn phosphorylation and aggregation, prevent mitochondrial dysfunction, attenuate oxidative stress, modulate autophagy primarily via AMPK activation, and prevent neurodegeneration and neuroinflammation (Paudel et al., 2020b). Several preclinical studies have been conducted to investigate the effects of metformin in PD models (Patil et al., 2014; Lu et al., 2016; Tayara et al., 2018; Katila et al., 2017). For instance, metformin reduced dopaminergic neuronal loss and motor deficits in methylphenidate tetrahydropyridine (MPTP)-induced mouse models of PD, and attenuated α-synuclein accumulation and mitochondrial dysfunction in rotenone-treated rats (Lu et al., 2016; Katila et al., 2017). These studies highlight metformin’s ability to modulate key pathological processes such as neuroinflammation, oxidative stress, and autophagic dysfunction, supporting its potential as a disease-modifying agent in PD.

In light of these findings, the present study aimed to investigate the possible protective role of metformin as added on therapy in PD based on these previous investigations.

## 2 Patients and methods

The study was conducted from June 2023 to August 2024 at the Neuro-Psychiatry Department of Tanta University’s Faculty of Medicine. Sixty participants from the Outpatient Clinic who met the inclusion criteria were included in the study. The National Research Ethics Committee of Tanta University Faculty of Medicine approved the study under license code (36264PR198/5/23). The study design and methodology adhered to the principles of the Helsinki Declaration and its 1964 revisions. Participants were informed that they could withdraw from the study at any time.

### 2.1 Inclusion criteria

Participants who were 50 years of age or older, male or female, had a diagnosis of PD, and receiving Levodopa/Carbidopa medication were eligible. Patients were diagnosed according to the Movement Disorder Society Clinical Diagnostic Criteria for Parkinson’s Disease (Postuma et al., 2015), which outline the key motor and non-motor symptoms required for a diagnosis, as well as exclusion criteria.

Regarding the age criterion, 50 years was selected based on epidemiological data (Mehanna et al., 2022; Ben-Shlomo et al., 2024) or the study’s objectives.

1. Epidemiological Justification: PD is predominantly diagnosed in individuals over 50 years old, with the incidence increasing significantly with age. This criterion aligns with the typical age of onset in most patient populations.
2. Study Design Considerations: By focusing on individuals 50 years and older, we aimed to reduce variability in disease onset and progression often observed in younger-onset cases, which may have distinct genetic and clinical profiles. This approach also allowed for a more homogenous participant pool, enhancing the reliability of our findings.

### 2.2 Exclusion criteria

Exclusion criteria included secondary parkinsonism, diabetes, cardiovascular diseases, atypical parkinsonian syndromes, prior stereotactic surgery for PD, senile tremors, Wilson’s disease, current cancer, as well as patients taking anti-inflammatory drugs. Pregnant and lactating females, individuals with a history of alcohol and/or drug addiction, and those with known allergies to the studied medications were also excluded.

### 2.3 Study design

**FIGURE 1:**
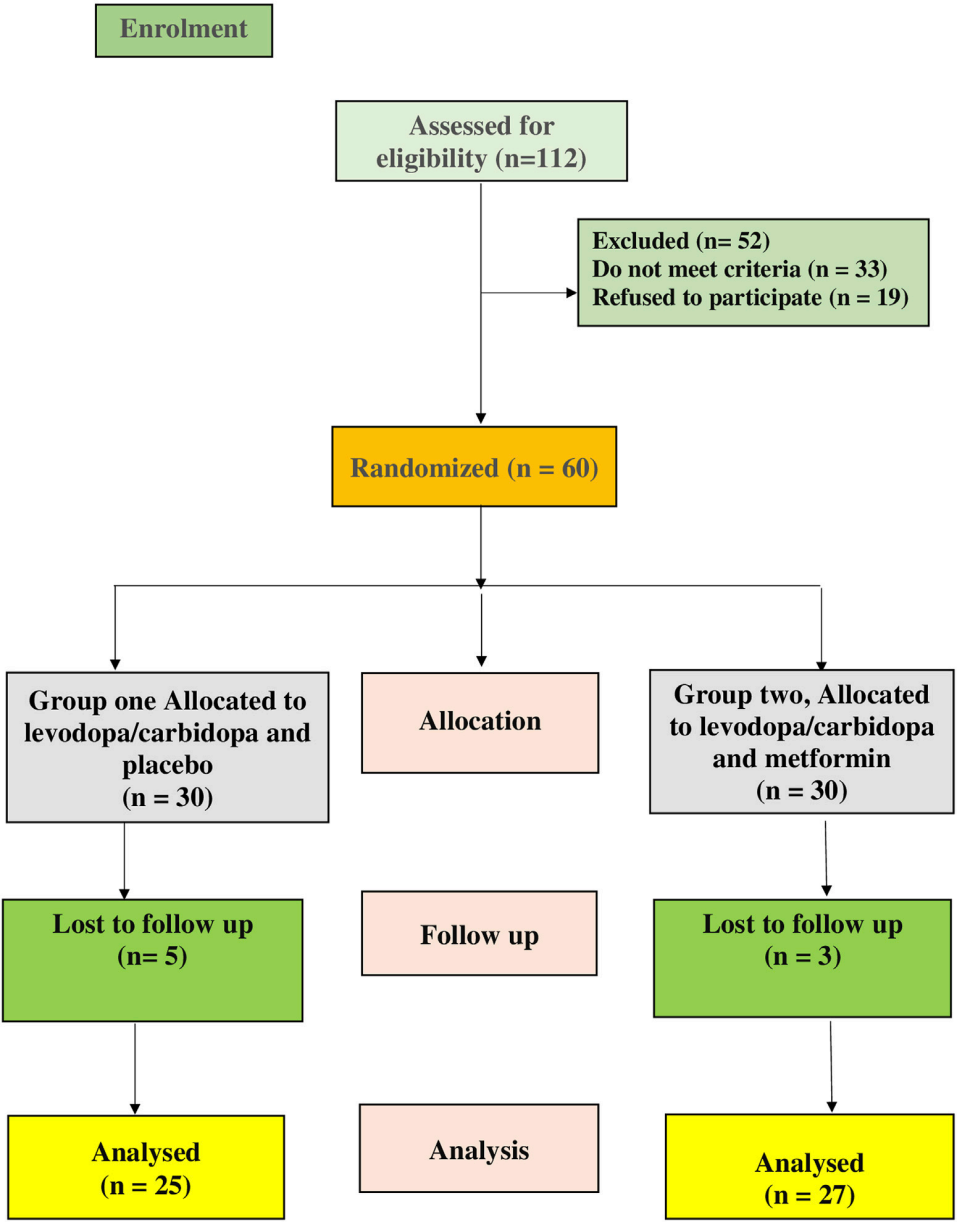
CONSORT diagram showing the flow of patients during the study.

This was a prospective, randomized, double-blind pilot clinical study aimed at determining the safety and efficacy of metformin in PD patients. The trial was registered on ClinicalTrials.gov with the identifier NCT05781711. Participants were randomly assigned to two groups (n = 30 each), as depicted in the CONSORT flow diagram in Figure 1. Metformin (El-Haggar et al., 2024) and levodopa/carbidopa (Khrieba et al., 2024) doses were based on earlier research. The dose of metformin used in our study (e.g., 1,000 mg/day in divided doses) was chosen based on both safety considerations and translational relevance, referencing prior clinical studies in non-diabetic neurological conditions (Abdelgaied et al., 2024; Halabitska et al., 2025). The 3-month follow-up period was selected based on several considerations. First, previous preclinical and clinical studies have shown that metformin exerts measurable effects on neuroinflammatory and neuroprotective markers—including TLR4, HMGB1, α-synuclein, and BDNF—within a similar or shorter duration. For example, in rodent PD models, metformin significantly modulated inflammatory pathways and mitochondrial markers within 4–8 weeks (Lu et al., 2016; Katila et al., 2017). Additionally, in human studies, 12 weeks of metformin treatment has been associated with significant changes in circulating cytokines (Halabitska et al., 2025; Banaszewska et al., 2011). From a clinical standpoint, a 3-month duration provides a practical balance between capturing early biological responses and maintaining high patient compliance in an elderly population that is often burdened by complex medication regimens and comorbidities.

Randomization was performed using random permuted blocks and a computer-generated random number sequence. Patients were required to discontinue all unnecessary medications, except for Levodopa/Carbidopa, for at least 2 weeks prior to trial participation.

Group 1: Control group (Levo-dopa group, n = 30) who received placebo and levodopa/carbidopa (250/25 mg) three times daily for 3 months (Sinemet^R^ tablets, Merck, Germany).
Group 2: Metformin group (n = 30) who received levodopa/carbidopa (250/25 mg) three times daily plus metformin 500 mg twice daily for 3 months (Glucophage^R^ tablets, Mina Pharm, Egypt).

### 2.4 Sample size calculation

No previous studies were available to estimate the actual effect size of metformin use on change in unified Parkinson disease rating scale (UPDRS). This study was constructed as a pilot study, as recommended by Sim and Lewis (2012), who proposed a sample size of at least 55 to adequately identify small to medium effect sizes and minimizing variability. The study used a randomised sample size of 30 patients per group, with an α-error of 0.05 (2-tailed) and a power of 0.80, with an adjustment for a 10% dropout rate.

### 2.5 Therapeutic assessment

#### 2.5.1 Primary outcome

The improvement in the Unified Parkinson’s Disease Rating Scale (UPDRS) was the primary outcome. The UPDRS was first introduced in 1987 at the “Recent Developments in Parkinson’s Disease” conference by a group of professionals in the field (Fahn, 1987). The UPDRS is designed to assess the signs and symptoms of Parkinson’s disease (PD). It can be administered across multiple patient encounters to track PD progression over time. The scale consists of 42 questions, some of which have multiple parts, as well as the Hoehn and Yahr Stage and the Schwab and England Activities of Daily Living Scale. It includes subscores for the following sections: “Mentation, Behavior, and Mood,” “Activities of Daily Living,” “Motor Examination,” and “Complications of Therapy,” along with an overall UPDRS score. Both the overall score and subscores are calculated by summing the numerical responses in the respective sections. The maximum possible UPDRS score is 199, reflecting the most severe level of disability due to PD, while the lowest score is 0, indicating the absence of PD signs and symptoms (Fahn, 1987).

#### 2.5.2 Secondary outcomes

Serum levels of biomarkers such as TLR-4, brain derived neurotropic factor (BDNF), HMGB1, and α-syn were evaluated as a secondary outcome measure to assess the therapeutic effects of drugs.

### 2.6 Study protocol

A neurologist evaluated the patients at baseline and 3 months after they started the medication. Patients were also questioned about drug adherence and potential adverse effects. Every 2 weeks, patients were contacted by phone to monitor their adherence to the study medication and report any side effects. All medications were administered orally. Both the type of treatment and the randomization process were kept blinded from both the patients and medical professionals. To assess patient adherence, the number of tablets remaining in each medication supply was counted. An unblinded pharmacist, who was not involved in outcome assessment, provided the study drugs to participants to ensure accurate therapy assignment. Blinding would only be broken by the responsible neurologist in the event of an emergency requiring knowledge of the current treatment. Once the blinding was broken, the patient would be withdrawn from the trial. Participants were also withdrawn if they discontinued the trial medication for seven consecutive days.

### 2.7 Sample collection

Ten milliliters (10 mL) of venous blood were drawn from the antecubital vein before the study began and 3 months after the intervention. The blood was carefully placed into test tubes, allowed to clot, and then centrifuged for 10 min at 4,500 g (Hettich Zentrifugen EBA 20). The serum was divided into two portions: the first was used for routine tests, and the second was stored at −80°C for biomarker analysis.

### 2.8 Biochemical analysis

A spectrophotometric kinetic approach was used to quantify the hepatic enzymes alanine aminotransferase (ALT) and aspartate aminotransferase (AST). Glycated hemoglobin (HbA1c) was measured for each patient before starting the trial. Measurements of serum creatinine (SCr) levels, a marker of renal function, were performed using the Jaffé reaction.

According to the instructions of the manufacturer (Sunred, Shanghai, China), commercially available enzyme-linked immunosorbent assay (ELISA) kits were used to measure serum levels of BDNF (catalogue no: 201-12-1303), TLR-4 (catalogue no: 201-12-0347), and α-syn (catalogue no: 201-12-1314), and HMGB-1 (catalogue no: 201-12-1636).

### 2.9 Statistical analysis

Prism 9 (GraphPad software, Inc., San Diego, CA, United States) was used to conduct the statistical analyses. The normal distribution of continuous variables was examined using the Shapiro-Wilk test. Significant differences within the group before and after therapy were found using Wilcoxon test for nonparametric data. To find significant variations between groups before and after therapy, unpaired Student's t-tests and Man Whitny test were performed for parametric and nonparametric data respectively. In terms of numbers, median, interquartile range and percentages, qualitative variables were provided, while quantitative values were expressed as mean and SD. On categorical data, the Chi-square test and fisher exact test were applied. All *p* values were two-tailed, with p < 0.05 considered statistically significant.

## 3 Results

### 3.1 Clinical and demographic characteristics

There were no statistically significant differences in baseline demographic data between the control and metformin groups as followed; age (*p* = 0.260), sex (*p* = 0.795), body mass index (*p* = 0.805), ALT (*p* = 0.827), AST (*p* = 0.905), SCr (*p* = 0.474), smoking (*p* = 0.273), duration of the disease (*p* = 0.526), and HbA1C (*p* = 0.276) (Table 1. Five patients were withdrawn from the control group because they developed progressive symptoms and required amantadine as an added-on therapy. Three patients were withdrawn from the metformin group because two of them were shifted to combined treatment and the remaining one did not attend to the hospital, accordingly the statistical analysis was performed per protocol to evaluate the biological and causal effects of the treatment as shown in (Figure 1).

**TABLE 1:** Clinical and demographic data in the two study groups.

Parameter	Control group (n = 30)	Metformin group (n = 30)	*p*-value
Age (year)	64.80 ± 6.63	66.97 ± 8.054	0.260
Sex (M/F)	15/15	14/16	0.795
BMI (kg/m^2^)	23.73 ± 1.081	22.99 ± 1.683	0.805
ALT (U/L)	21.83 ± 6.909	21.30 ± 5.459	0.741
AST (U/L)	25.37 ± 6.599	23.90 ± 7.331	0.418
SCr (mg/dL)	0.978 ± 0.166	0.948 ± 0.152	0.474
Smoking (n, %)	8 (26.6%)	12 (40%)	0.273
Duration of disease (years)	2.253 ± 0.924	2.413 ± 1.016	0.526
HA1c (%)	5.376 ± 0.246	5.287 ± 0.370	0.276

Data are expressed as mean ± SD, percentage and numbers, M, male; F, female; BMI, body mass index; ALT, Alanine amino-transferase; AST, Aspartate amino-transferase; SCr, Serum creatinine; HA1c, glycated haemoglobin. Significance at (*p* < 0.05). Differences between groups for each characteristic were tested for significance using unpaired t-test for continuous data and Chi square test for categorial data.

### 3.2 Analysis of unified Parkinson’s disease rating scale and its subscale in the two study groups

Table 2 demonstrated no significant difference in baseline values between the two groups using Man Whitney test (*p* > 0.05).

**TABLE 2:** Analysis of unified Parkinson’s disease rating scale and its subscale in the two study groups.

Character	Control group (n = 25)			Metformin group (n = 27)			^*b*^ *p* value	Effect size
	Before therapy	After therapy	^a^ *p* value	Before therapy	After therapy	^a^ *p* value	After therapy	Rank-biserial correlation coefficient (r)
Mentation, Behavior and Mood	12 (10-14.5)	11 (10-12.5)	0.603	13 (12-14)	10 (9-12)	0.005	0.195	0.208
Activities of Daily Living	42 (38.5-44)	39 (35-41)	0.215	41 (38-45)	36 (30-44)	0.039	0.441	0.125
Motor Examination	88 (52-96)	84 (38-90.5)	0.537	90 (63-94)	72 (57-86)	0.011	0.426	0.130
Complications of Therapy	16 (9.5-18)	13 (6.5-17)	0.144	17 (14-20)	12 (7-16)	0.007	0.705	0.062
UPDRS total score	141 (115-164)	139 (101-151.5)	0.278	152 (129-166)	128 (98-151)	0.001	0.515	0.106
Schwab and England Activities of Daily Living Scale	60 (50-70)	70 (30-90)	0.393	50 (40-60)	80 (50-90)	0.0002	0.137	0.238
Modified Hoehn and Yahr Staging	3 (2-3)	2 (1.5-3)	0.263	3 (2.5-4)	1 (1-3)	0.003	0.082	0.278

Data are expressed median, and interquartile range. Control group: patients received levodopa/carbidopa and placebo for three months, Metformin group: patients received levodopa/carbidopa for three months plus metformin for three months. UPDRS, Unified Parkinson's Disease Rating Scale. (a) within group comparison using Wilcoxon test, (b) between group comparison using Man Whitney test, Significance at (*p* < 0.05).

Regarding control group, within group comparison, Wilcoxon test showed that there was no significant decrease in the median value for the following parameters when compared to baseline as followed: Mentation, Behaviour and Mood [12 (10–14.5) versus 11 (10–12.5), *p* = 0.603], Activities of Daily Living [42 (38.5–44) versus 39 (35–41), *p* = 0.215], Motor Examination [88 (52–96) versus 84 (38–90.5), *p* = 0.537], Complications of Therapy [16 (9.5–18) versus 13 (6.5–17), *p* = 0.144], and UPDRS total score [141 (115–164) versus 139 (101–151.5), *p* = 0.278]. Additionally, Wilcoxon test showed that there was no significant change in median value of the following parameters: Schwab and England Activities of Daily Living Scale [60 (50–70) versus 70 (30–90), *p* = 0.393], and Modified Hoehn and Yahr Staging [3 (2–3) versus 2 (1.5–3), *p* = 0.263] (Table 2).

Regarding metformin group, within group comparison, Table 2 revealed that the following parameters were significantly reduced by using Wilcoxon test when compared to their baseline values as followed: Mentation, Behavior and Mood [13 (12–14) versus 10 (9–12), *p* = 0.005], Activities of Daily Living [41 (38–45) versus 36 (30–44), *p* = 0.039], Motor Examination [90 (63–94) versus 72 (57–86), *p* = 0.011], Complications of Therapy [17 (14–20) versus 12 (7–16), *p* = 0.007], and UPDRS total score [152 (129–166) versus 128 (98–151), *p* = 0.001]. Also, Wilcoxon test showed that there was a significant change in the following parameters: Schwab and England Activities of Daily Living Scale [50 (40–60) versus 80 (50–90), *p* = 0.0002], and Modified Hoehn and Yahr Staging [3 (2.5–4) versus 1 (1–3), *p* = 0.003] (Table 2).

Between group comparison, Man Whitney test showed that there were no statistically significant changes in UPDRS and its subscale after 3 months of intervention between the two groups, as followed: Mentation, Behaviour and Mood (*p* = 0.195), Activities of Daily Living (*p* = 0.441), Motor Examination (p = 0.426), Complications of Therapy (*p* = 0.705), UPDRS total score (*p* = 0.515), Schwab and England Activities of Daily Living Scale (*p* = 0.137), and Modified Hoehn and Yahr Staging (*p* = 0.08) (Table 2).

### 3.3 Analysis of serum biomarkers in the two study groups

Table 3 demonstrated no statistically significant difference in baseline values between the two groups using Man Whitney test (*p* > 0.05).

**TABLE 3:** Analysis of serum biomarkers in the two study groups.

Character	Control group (n=25)			Metformin group (n=27)			^*b*^ *p* value	Effect size
	Before therapy	After therapy	^a^ *p* value	Before therapy	After therapy	^a^ *p* value	After therapy	Rank-biserial correlation coefficient (r)
α-synuclein (ng/ml)	70 (60.35-90)	68.7 (35.40- 84.59)	0.107	73 (60-81)	54.8 (26.5-66)	0.0005	0.03	0.331
BDNF (ng/ml)	4.7 (3.4-6.355)	5.2 (4.24- 8.91)	0.074	4.16 (3.28-4.7)	8.28 (5.25- 9.62)	0.003	0.02	0.367
HMGB-1 (ng/ml)	155 (143-173.5)	150 (143-173.5)	0.610	151 (103-176)	120 (75-160)	0.001	0.03	0.336
TLR4 (ng/ml)	10.48 (9.27-11.22)	9.5 (6.7-10.67)	0.140	9.6 (8.47-10.96)	5.36 (3.62-10.62)	0.0008	0.04	0.322

Data are expressed as median and interquartile range, Significance at (*p* < 0.05)**.** Control group: patients received levodopa/carbidopa and placebo for three months, Metformin group: patients received levodopa/carbidopa for three months plus metformin for three months. Brain derived neurotropic factor (BDNF), toll like receptor 4 (TLR-4), high mobility group box protein 1(HMGB-1). (a) within group comparison using Wilcoxon test, (b) between group comparison using Man Whitney test, Significance at (*p* < 0.05).

Regarding control group, within group comparison, Wilcoxon test demonstrated that there was no significant change in median value of the following parameters when compared to baseline as followed: α-syn [70 (60.35–90) versus 68.7 (35.40–84.59), *p* = 0.107], HMGB-1 [155 (143–173.5) versus 150 (143–173.5), *p* = 0.610], TLR-4 [10.48 (9.27–11.22) versus 9.5 (6.7–10.67), *p* = 0.140], and BDNF [4.7 (3.4–6.355) versus 5.2 (4.24–8.91), *p* = 0.074].

Regarding metformin group, within group comparison by Wilcoxon test, Table 3 revealed that the following parameters were significantly reduced when compared with their baseline values as followed: α-syn [73 (60–81) versus 54.8 (26.5–66), *p* = 0.0005], HMGB-1 (151 (103–176) versus 120 (75–160), *p* = 0.001], and TLR-4 [9.6 (8.47–10.96) versus 5.36 (3.62–10.62), *p* = 0.0008], as well as a significant increase in BDNF [4.16 (3.28–4.7) versus 8.28 (5.25–9.62), *p* = 0.0003].

Between group comparison, Man Whitney test showed that there were statistically significant changes in all studied markers after 3 months of intervention, as followed: α-syn (*p* = 0.03), TLR-4 (*p* = 0.04), BDNF (*p* = 0.02), and HMGB-1 (*p* = 0.03).

### 3.4 Analysis of drug-related adverse effects between the groups

Table 4 showed that there were no significant differences between the studied groups in terms of side effects as followed: nausea (*p* = 0.844), vomiting (*p* = 0.705), diarrhea (*p* = 0.669), hypotension (*p* = 0.278), delusions (*p* = 0.705), and abdominal pain (*p* = 0.423).

**TABLE 4:** Comparison of drug-related adverse effects between the groups.

Side effect	Control group (n = 25)	Metformin group (n = 27)	*p* value
Nausea	5	6	0.844
Diarrhoea	2	4	0.669
Vomiting	3	5	0.705
Hypotension	5	9	0.278
Delusions	3	5	0.705
Abdominal pain	5	8	0.423

Control group: patients received levodopa/carbidopa for 3 months, metformin group: patients received levodopa/carbidopa for 3 months plus metformin for 3 months. Data were presented as numbers. Significance at (p < 0.05) using Chi square or fisher exact test as appropriate.

## 4 Discussion

To our knowledge, this is the first clinical research to investigate the neuroprotective role of metformin in PD and explore its mechanistic pathways in this neurodegenerative disorder.

Drug repurposing, also known as drug repositioning, is a promising approach for identifying new therapeutic uses for already approved medications. This strategy has demonstrated success in managing various conditions, such as PD, depression, non-alcoholic fatty liver disease, ulcerative colitis, breast cancer, inflammatory disorders, and colorectal cancer (Jarada et al., 2020; Pushpakom et al., 2019; Aldossary et al., 2024; Alarfaj et al., 2024; Alarfaj et al., 2023; Shawky et al., 2022; El-Haggar et al., 2022).

In the current study, the metformin group significantly decreased UPDRS and its subscale when compared to their baseline values. Levodopa/carbidopa is the cornerstone in the management of patients with PD. Adding metformin to the standard therapy reduced UPDRS scores; however, the change was not statistically significant compared to monotherapy. Although the metformin group showed significant changes in biomarkers, the differences in total UPDRS scores between the two groups did not reach statistical significance. Potential reasons for non-significant changes in UPDRS may include short follow-up period as the 3-month duration of the study may have been insufficient to observe significant clinical symptom improvements, as motor symptoms often progress slowly and may require a longer time to respond to interventions. The UPDRS, while widely used, may not be sensitive enough to detect subtle or early improvements in motor symptoms, particularly over a short duration. This limitation could obscure potential clinical benefits associated with biomarker changes. Also, individual variability such as differences in disease severity, progression rates, and response to treatment among participants could contribute to variability in UPDRS outcomes, potentially diluting the statistical significance. The disconnection between biomarkers and clinical symptom changes may also due to biomarker lag effect as improvements in biomarkers may precede observable clinical symptom changes, reflecting underlying disease-modifying effects that require more time to translate into functional improvements. Finally, complex pathophysiology of PD as PD involves multifactorial mechanisms, and changes in specific biomarkers may not directly correspond to symptomatic relief due to compensatory or unrelated pathways affecting clinical outcomes.

The current study demonstrated that the metformin group significantly reduced serum α-synuclein levels compared to both baseline and the control group. These findings are consistent with other research (Katila et al., 2017; Saewanee et al., 2021). According to Pérez-Revuelta et al., metformin reduces levels of Ser-129 phosphorylated α-syn by activating mammalian target of rapamycin (mTOR)-dependent protein phosphatase 2A (Pérez-Revuelta et al., 2014). By reducing lipid peroxidation, Ozbey et al. demonstrated that metformin decreased the levels of α-syn in rotenone-induced dopaminergic neurotoxicity (Ozbey et al., 2020). Metformin, acting independently of the pro-survival kinase and without stimulating the autophagic response, restored AMPK activity and reduced the *in vitro* neurotoxicity associated with α-synuclein overexpression (Dulovic et al., 2014). AMPK-dependent protection against extracellular α-syn was also demonstrated in the rat neuron-like pheochromocytoma cell line (PC12) (Dulovic et al., 2014; Jardim et al., 2018).

Several studies have established a substantial association between changes in blood biomarkers and clinical outcomes in PD, giving compelling evidence for their potential involvement in disease progression monitoring. Stewart et al. found a strong correlation between α-syn levels and the UPDRS. They also observed that cerebrospinal fluid (CSF) α-syn levels increased over approximately 2 years of disease progression in the Deprenyl and Tocopherol Antioxidative Therapy of Parkinsonism (DATATOP) cohort. These findings suggest that α-synuclein levels are associated with disease severity and clinical outcomes (Stewart et al., 2015; Stewart et al., 2014).

Ju-Hee Kang and colleagues developed a multivariate logistic regression (MLGR) model to examine the association between CSF biomarkers and PD diagnosis. As an initial step, they conducted a bivariate analysis of each CSF biomarker and PD clinical features, adjusting for confounders such as age, sex, and education. This analysis revealed significant associations between CSF T-tau (P = 0.02), P-tau-181 (P = 0.005), and α-syn (P = 0.04) with PD diagnosis (Kang et al., 2013). Also, Longitudinal changes in α-syn species reflect PD progression (Majbour et al., 2016).

The current study also revealed a significant decrease in the serum levels of TLR-4 and HMGB-1 in the metformin group compared to both their baseline values and the control group. These findings are consistent with previous studies (Qu and Qu, 2019; Alomar et al., 2021; Liu et al., 2024). It has also been reported that HMGB1 and TLR-4 expression levels were higher in the peripheral blood of patients with PD compared to healthy volunteers. PD patients with poor treatment outcomes exhibited significantly higher levels of HMGB1 and TLR-4 expression than those with stable treatment outcomes. Elevated HMGB1 and TLR-4 expression levels were observed in patients at more advanced stages of PD, and patients with a disease duration longer than 4 years showed significantly higher expression levels of HMGB1 and TLR-4 than those with a disease duration of less than 4 years (Yang et al., 2018b). High expression of the HMGB1–TLR-4 axis is crucial for the diagnosis and treatment of PD and is strongly associated with the onset, progression, treatment efficacy, staging, and duration of the disease (Yang et al., 2018b).

Alomar et al. reported that metformin suppresses TLR-4/NF-κB expression and glutamate excitotoxicity (Alomar et al., 2021). Metformin has also been shown to inhibit acute neutrophil activation and recruitment through an AMPK-dependent mechanism (Ashayeri Ahmadabad et al., 2024). Furthermore, it has been shown that intercellular adhesion molecule 1 (ICAM-1) expression is regulated by NF-κB, and metformin reduces ICAM-1 expression, which in turn reduces TLR-4 (Liu et al., 2021). Co-treatment with the natural HMGB1 inhibitor Glycyrrhizin exerts neuroprotection and reverses PD-like pathology (Ren et al., 2022). Metformin directly binds the alarmin HMGB1 and inhibits its proinflammatory activity (Horiuchi et al., 2017). Metformin also alleviates HMGB1-mediated oxidative stress through the mTOR pathway in experimental periodontitis (Sun et al., 2023). Metformin ameliorates doxorubicin-induced cardiotoxicity by targeting the HMGB1/TLR4/NLRP3 signaling pathway in mice (Alzokaky et al., 2023).

The current study demonstrated that metformin combined with levodopa/carbidopa therapy significantly increased BDNF compared to both the baseline value and the control group. These findings are consistent with previous studies (Katila et al., 2017; Houshmand et al., 2019). According to research by Katila et al., metformin boosts neurotrophic factor levels in the methylphenidate-tetrahydropyridine (MPTP) animal model of PD (Katila et al., 2017). According to Miyoshi et al., significant behavioral improvements were observed following the administration of a neurotrophic factor, when comparing the levodopa dose-response before and after therapy (Miyoshi et al., 1997). Additionally, parkinsonian animals treated with levodopa/carbidopa alone experienced side effects as dystonias, dykinesias, vomiting, and stereotypical movements (Miyoshi et al., 1997). These levodopa-induced side effects were greatly decreased by the administration of neurotrophic factor along with levodopa/carbidopa, with a >90% reduction in adverse reactions observed at the mid-levodopa/carbidopa dose level (250 mg levodopa-25 mg carbidopa) (Miyoshi et al., 1997). Thus, combining metformin, a neurotrophic factor upregulator, with levodopa/carbidopa treatment may be therapeutically beneficial in treating parkinsonism by improving functional response and reducing adverse effects of levodopa/carbidopa. Additionally, by increasing BDNF and generating neurotrophic factors, metformin-induced AMPK activation promotes remyelination. According to studies by Paintlia et al., metformin treatment enhanced the production of BDNF in rats with experimental autoimmune encephalomyelitis (EAE) (Paintlia et al., 2013). A previous research regarding metformin suggests that metformin enhances neurogenesis by stimulating an atypical Protein kinase C-CREB-binding protein (PKC-CBP) pathway (Wang et al., 2012), which play fundamental role in neurodevelopment, neuroprotection, and synaptic plasticity (Sakamoto et al., 2011).

Since each drug is metabolized by a distinct isoenzyme, it is noteworthy that no pharmacokinetic interactions between metformin and levodopa/carbidopa have been documented (Gong et al., 2012; Contin and Martinelli, 2010). Furthermore, there were no significant differences in the baseline clinical data between the patients. Since these differences cannot explain the variations in therapeutic responses between the groups, the therapeutic benefits are most likely due to the effects of the combined treatments.

Despite the promising results of the current study, some studies on type 2 diabetes mellites patients with high doses and long-term metformin therapy reported that metformin may increase the risk of PD by inducing hyperhomocysteinemia and deficiency of folate and vitamin B12 (Infante et al., 2021; Alrouji et al., 2024c; Tiwari et al., 2023). Long-term metformin use has been associated with reduced vitamin B12 absorption, potentially leading to deficiency (Infante et al., 2021). This condition can cause neurological and hematological complications, which are particularly concerning in populations already at risk for neurodegenerative diseases. We recommend routine monitoring of vitamin B12 levels in long-term users to mitigate these risks. Thus, further studies are required to validate these conflicting results. A secondary effect of vitamin B12 deficiency is the elevation of homocysteine levels, which may contribute to vascular complications (Mohan et al., 2023). While the clinical relevance of this in the context of metformin use is still under investigation, we acknowledge this as a potential risk and suggest that future studies should explore its implications more thoroughly.

Furthermore, the variability in metabolic rates among participants could affect biomarker levels, particularly those associated with energy expenditure and metabolic processes. While we did not measure basal metabolic rates directly, we acknowledge this limitation and suggest it as a point for further investigation in future studies. Also, Differences in dietary intake, including macronutrient composition and caloric consumption, could influence certain biomarkers. Dietary habits that may influence biomarkers in Parkinson’s disease (PD) include a generally healthy diet, the protein-restricted diet (PRD), the ketogenic diet (KD), the Mediterranean diet (MD), and the Mediterranean-DASH Intervention for Neurodegenerative Delay (MIND) diet (Knight et al., 2022). Although dietary habits were not controlled in this study, we have emphasized the need to account for these variables in subsequent research to reduce their confounding effects.

Moreover, there were some limitations that included short duration period, small sample size, and lack of different doses of metformin to determine the optimum dose. We recognize that certain effects of metformin, particularly those involving neuroprotective mechanisms or disease-modifying properties, may take longer to manifest. A longer follow-up would provide a more comprehensive evaluation of treatment outcomes and help assess sustained effects on PD progression. To address this limitation, we have emphasized the need for future studies to incorporate follow-up periods extending beyond 6 months to 1 year or more. Such studies could provide a more detailed understanding of metformin’s long-term impact on PD motor symptoms, biomarkers, and overall disease trajectory.

While our current study focused on key biomarkers related to inflammation (TLR-4, HMGB-1), neurotrophic support (BDNF), and protein aggregation (α-synuclein), we recognize the value of broadening this panel. Future studies will consider markers of oxidative stress, mitochondrial function, and synaptic integrity to provide a more comprehensive understanding of metformin’s mechanisms in PD.

The present study is a monocentric study performed on a Middle East population. Accordingly, the benefit observed in this study should be verified in multicenter studies and in other ethnic groups, we recommend large scale and different doses clinical trials to validate these results. It would have been advisable to assess lipid profile, vitamin B12, and blood glucose also at the end of the study. Furthermore, conducting longer-term studies to assess whether biomarker improvements eventually result in clinical symptom changes. Investigating additional factors, such as participant heterogeneity and interaction between biomarkers and clinical features, to better understand the biomarker-symptoms relationship.

## 5 Conclusion

While no significant differences in UPDRS scores were observed between the metformin and control groups, trends in biomarker changes suggest a potential impact of adjunctive metformin use on the underlying pathophysiology of PD. Metformin could alleviate inflammatory and oxidative stress biomarkers by modulation of HMGB-1/TLR-4, and α-syn signaling pathways. Further clinical trials are required to confirm the benefits and safety profile of metformin in PD.

## Data Availability

The raw data supporting the conclusions of this article will be made available by the authors, without undue reservation.
